# Aortic Dysfunction in Mitral Regurgitation Due to Floppy Mitral Valve/Mitral Valve Prolapse

**DOI:** 10.1055/s-0038-1669417

**Published:** 2018-09-12

**Authors:** Hatem Mowafy, Scott Lilly, David A. Orsinelli, Gregory Rushing, Juan Crestanello, Konstantinos Dean Boudoulas

**Affiliations:** 1 Division of Cardiovascular Medicine, Department of Medicine, The Ohio State University, Columbus, Ohio; 2 Critical Care Department, Faculty of Medicine, Cairo University, Cairo, Egypt; 3 Division of Cardiac Surgery, Department of Surgery, The Ohio State University, Columbus, Ohio

**Keywords:** floppy mitral valve, mitral valve prolapse, aorta, distensibility

## Abstract

**Background** Floppy mitral valve/mitral valve prolapse (FMV/MVP), a heritable disorder of connective tissue, often leads to mitral regurgitation (MR) and is the most common cause for mitral valve surgery in developed countries. Connective tissue disorders may affect aortic function, and a stiff aorta may increase the severity of MR. Aortic function, however, has not been studied in FMV/MVP with MR.

**Methods** A total of 17 patients (11 men, 6 women) with FMV/MVP and significant MR were compared with 20 controls matched for age and gender. Aortic diameters (AoD) were measured from left ventriculograms at 2 and 4 cm above the aortic valve. Aortic pressures were measured directly using fluid-filled catheters. Aortic distensibility was calculated using the formula: 2(systolic AoD—diastolic AoD)/(diastolic AoD x pulse pressure).

**Results** Aortic distensibility was significantly lower in FMV/MVP compared with control at 2 cm above the aortic valve (1.00 ± 0.19 versus 3.78 ± 1.10 10 ^−3^ mm Hg ^−1^ , respectively; *p* = 0.027) and 4 cm above the aortic valve (0.89 ± 0.16 versus 3.22 ± 0.19 10 ^−3^ mm Hg ^−1^ , respectively; *p* = 0.007). FMV/MVP patients had greater left ventricular (LV) end-systolic (88 ± 72 mL versus 35 ± 15 mL, *p* = 0.002) and end-diastolic (165 ± 89 mL versus 100 ± 41 mL, *p* = 0.005) volumes, and lower LV ejection fraction, compared with control (50 ± 12% versus 57 ± 6%, *p* = 0.034).

**Conclusion** Aortic distensibility is decreased (consistent with a stiff aorta) in patients with FMV/MVP and MR. A stiff aorta may increase the severity of MR. Thus, abnormal aortic function, which also deteriorates with age, may play an important role in the natural history of MR due to FMV/MVP.

## Introduction

Floppy mitral valve/mitral valve prolapse (FMV/MVP) is considered to be a heritable disorder of connective tissue and has a prevalence of 2 to 3% in the general population. 1 2 3 FMV/MVP is most commonly transmitted by an autosomal dominant inheritance with a variable degree of penetration. 1 4 5 6 The term FMV comes from surgical and pathologic studies, and refers to the expansion of the area of the mitral valve leaflets with elongated chordae tendineae and frequently dilated mitral annulus. MVP is a nonspecific finding and can be produced by several mechanisms such as papillary muscle rupture or dysfunction, left ventricular (LV) wall motion abnormalities, small LV size, hyperdynamic LV function, and others. Thus, FMV is the central issue in primary MVP and for these reasons it should be used in all cases of primary MVP. 7 Patients with FMV/MVP over the years may develop severe mitral regurgitation (MR) requiring surgery or other interventional therapy. FMV/MVP is the most common cause for mitral valve surgery in developed countries. 1 2 3 6

The clinical phenotype of FMV/MVP is widely heterogeneous, ranging from a benign clinical course with a normal life expectancy to adverse outcomes with significant morbidity and mortality, mostly related to MR and its complications. As a connective tissue disorder, FMV/MVP may be associated with structural abnormalities of other organs. 1 2 8 9 Glesby and Pyeritz emphasized some of these findings using the acronym MASS, indicating involvement of the **m** itral valve, **a** orta, **s** keleton and **s** kin. 9 Occasionally, FMV/MVP may be part of a well-defined syndrome of a heritable connective tissue disorder such as the Marfan syndrome, Ehlers–Danlos syndrome, polycystic kidney disease, and others. 9 10 11 Although involvement of the aorta has been suggested in FMV/MVP patients, and a stiff aorta may increase the severity of MR, there is a lack of information related to function of the aorta in patients with FMV/MVP. 9 10 11 12 The present study was undertaken to evaluate aortic function in patients with FMV/MVP and significant MR.

## Methods

### Study Population

Seventeen patients (11 men, 6 women) with isolated FMV/MVP and significant MR requiring surgery were studied. The severity of MR was determined by left ventriculogram and by two-dimensional transthoracic echocardiogram using several methods including area of regurgitant jet compromising the left atrium, vena contracta, effective regurgitant orifice, regurgitant volume, regurgitant fraction, and/or pulmonary vein systolic flow reversal. All patients had chronic symptomatic MR requiring surgical intervention in which the cardiologist, interventional cardiologist, and cardiothoracic surgeon were in agreement. The diagnosis of FMV/MVP was established preoperatively by echocardiography and by direct inspection of the mitral valve during surgery. FMV was also established with histopathologic examination in 15 of the patients. All patients had isolated FMV/MVP and those with a known heritable connective tissue disorder were excluded prior to entry into the study. Heritable connective tissue disorders were excluded by taking a detailed personal and family history. Twenty individuals (9 men, 11 women) matched for age and gender without FMV/MVP or MR were used as control.

All studied subjects (patients and controls) underwent selective coronary arteriography and left ventriculography. Coronary arteriography and left ventriculography were performed as part of a preoperative evaluation in patients with FMV/MVP and to define coronary anatomy in the control group as part of their clinical evaluation. None of the studied individuals had significant coronary artery disease (defined as > 40% stenosis in any of the coronary arteries). The protocol of the study was approved by the Institutional Review Board (IRB); individual consent was waived due to the retrospective nature of the study.

### Assessment of Aortic Function

**Fig. 1:**
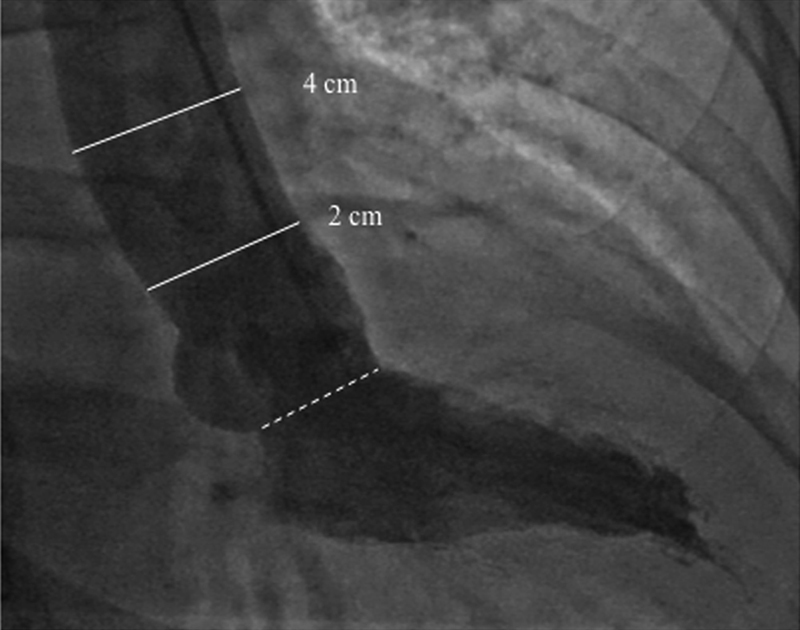
Left ventriculogram in the right anterior oblique projection. Aortic diameters were measured at end-systole and end-diastole 2 cm and 4 cm above the aortic valve (dotted line).

Aortic distensibility was determined from changes in the aortic diameter (systolic minus diastolic) and aortic pressure (systolic minus diastolic), as described previously. 13 14 15 16 In the cardiac catheterization laboratory, aortic pressures were measured directly using fluid-filled catheters prior to the injection of contrast medium. Left ventriculogram was performed in the right anterior oblique projection using the General Electric Innova system (General Electric Healthcare); frame-by-frame analysis was then performed to define LV end-systolic and end-diastolic frames. Aortic diameters were then measured at end-systole and end-diastole 2 cm and 4 cm above the aortic valve ( Fig. 1 ); measuring aortic distensibility at two different levels of the aorta was performed to further strengthen the results, as described previously. 16 LV end-diastolic and end-systolic volumes were also measured, as well as ejection fraction was determined.

Aortic distensibility was measured using the formula 16 :



### Statistical Analysis

Descriptive data are shown as mean ± 1 standard deviation. To determine statistical significance between the two groups Fisher's exact test, chi-square or the Student's *t* -test were used where appropriate. A *p* -value < 0.05 was considered as statistically significant.

## Results

Baseline demographic and clinical parameters are shown in Table 1 . As compared with control, patients with FMV/MVP had greater LV end-systolic (88 ± 72 mL versus 35 ± 15 mL; *p* = 0.002) and end-diastolic (165 ± 89 mL versus 100 ± 41 mL; *p* = 0.005) volumes, and lower LV ejection fraction compared with control (50 ± 12% versus 57 ± 6%; *p* = 0.034). Aortic pressures (systolic, diastolic, mean) and pulse pressure (systolic minus diastolic) were not statistically significant between the two groups.

**Table 1:** Baseline characteristics and clinical parameters in patients with FMV/MVP and control

	FMV/MVP ( *n* = 17)	Control ( *n* = 20)	*p* Value
Age (years)	55 ± 14	54 ± 18	0.887
Gender			0.385
Male, *n* (%)	11 (65)	9 (45)	
Female, *n* (%)	6 (35)	11 (55)	
Heart rate (bpm)	77 ± 13	81 ± 15	0.404
Systolic blood pressure (mm Hg)	132 ± 28	118 ± 24	0.103
Diastolic blood pressure (mm Hg)	78 ± 16	70 ± 10	0.064
Mean blood pressure (mm Hg)	99 ± 20	92 ± 16	0.240
Pulse pressure (mm Hg)	54 ± 15	48 ± 23	0.378
LVEDP (mm Hg)	18 ± 9	14 ± 5	0.077
LVESV (mL)	88 ± 72	35 ± 15	0.002
LVEDV (mL)	165 ± 89	100 ± 41	0.005
LVEF (%)	50 ± 12	57 ± 6	0.034
Severe mitral regurgitation, *n* (%)	14 (82)	0 (0)	< 0.0001

Abbreviations: FMV/MVP, floppy mitral valve/mitral valve prolapse; LVEDP, left ventricular end-diastolic pressure; LVEDV, left ventricular end-diastolic volume; LVEF, left ventricular ejection fraction; LVESV, left ventricular end systolic volume.

**Fig. 2:**
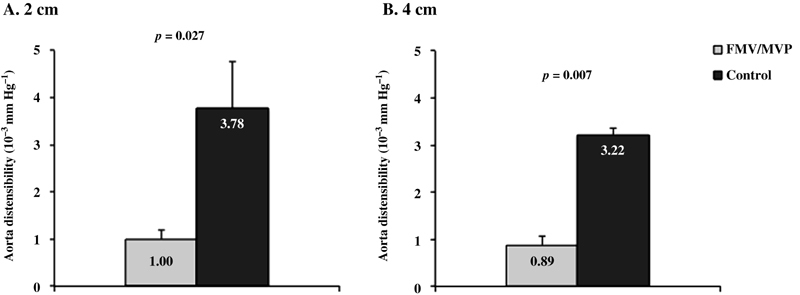
Aortic distensibility at 2 cm ( **A** ) and 4 cm ( **B** ) above the aortic valve are shown in patients with floppy mitral valve (FMV)/mitral valve prolapse (MVP) and in the control group.

Table 2 shows aortic diameters in systole and diastole, and aortic distensibility in FMV/MVP and control groups. Systolic and diastolic diameters at both levels of the aorta (2 cm and 4 cm above the aortic valve) were not statistically different between the two groups. Change of the aortic diameter from diastole to systole was significantly less in patients with FMV/MVP compared with control group at 2 cm above the aortic valve (0.06 ± 0.04 versus 0.16 ± 0.08 cm, respectively; *p* < 0.0005) and 4 cm above the aortic valve (0.06 ± 0.03 versus 0.15 ± 0.07 cm, respectively; *p* < 0.0001). Aortic distensibility was also significantly lower in patients with FMV/MVP compared with control group at 2 cm above the aortic valve (1.00 ± 0.19 versus 3.78 ± 1.10 10 ^−3^ mm Hg ^−1^ , respectively; *p* = 0.027; Fig. 2A ) and 4 cm above the aortic valve (0.89 ± 0.16 versus 3.22 ± 0.19 10 ^−3^ mm Hg ^−1^ , respectively; *p* = 0.007; Fig. 2B ).

**Table 2:** Aortic diameters and distensibility in patients with FMV/MVP and control

	FMV/MVP ( *n* = 17)	Control ( *n* = 20)	*p* Value
**2 cm above aortic valve**			
Aortic systolic diameter (cm)	2.72 ± 0.40	2.97 ± 0.61	0.163
Aortic diastolic diameter (cm)	2.66 ± 0.40	2.81 ± 0.60	0.376
Change in aortic diameter (cm)	0.06 ± 0.04	0.16 ± 0.08	< 0.0005
Aortic distensibility a	1.00 ± 0.19	3.78 ± 1.10	0.027
**4 cm above aortic valve**			
Aortic systolic diameter (cm)	2.78 ± 0.42	3.00 ± 0.61	0.227
Aortic diastolic diameter (cm)	2.72 ± 0.42	2.85 ± 0.63	0.482
Change in aortic diameter (cm)	0.06 ± 0.03	0.15 ± 0.07	< 0.0001
Aortic distensibility a	0.89 ± 0.16	3.22 ± 0.19	0.007

Abbreviation: FMV/MVP, floppy mitral valve/mitral valve prolapse.

Note: All data shown represents mean ± standard deviation except for aortic distensibility that is shown as mean ± standard error of the mean.

a Units for aortic distensibility are 10 ^−3^ mm Hg ^−1^ .

## Discussion

FMV/MVP can result in significant MR and if not treated can lead to heart failure. In symptomatic patients with FMV/MVP and severe MR, mitral valve surgery is the treatment of choice. FMV/MVP is the most common cause for mitral valve surgery in developed countries today. 1 2 3 6

FMV/MVP is considered to be a heritable disorder of connective tissue. 1 4 5 6 Histopathologic characteristics in the leaflets of the mitral valve in patients with FMV/MVP include collagen dissolution and disruption in the pars fibrosa, and replacement of the dense collagenous fibrosa by loose myxomatous connective tissue; similar histologic changes are found in the chordae tendineae. 7 17 18 19

The present study demonstrated that aortic distensibility in the ascending aorta is decreased in patients with FMV/MVP and significant MR. Although aortic dysfunction may precipitate the progression of MR, up to this point there has been no information related to aortic function in patients with FMV/MVP. 1 2 It is possible that subtle histologic changes of the aortic wall may be present contributing to aortic dysfunction in these patients. 1 2 8 9 10 11 12 Preliminary studies from our laboratory have shown that patients with FMV/MVP and severe MR requiring surgery have polymorphisms of matrix metalloproteinases that alter collagen synthesis and degradation. Whether these findings are related to structural abnormalities and/or functional changes of the aortic wall remains to be defined.

The patients with FMV/MVP in this study had significant symptomatic MR. It is known that MR is associated with increased sympathetic activity and decrease in forward stroke volume, which both may decrease vasa-vasorum blood flow to the outer aortic wall of the ascending aorta. 20 21 22 Previous studies at The Ohio State University and other investigations have shown that a decrease in vasa-vasorum flow is associated with decreased aortic distensibility. 23 24 Further, it is known that patients with FMV/MVP even without MR may have high adrenergic activity and hyper-response to adrenergic stimulation. 25 26 Previous studies from our laboratory have shown that patients with FMV/MVP have β1-adrenergic receptor polymorphisms that enhance adrenergic activity. 27 High adrenergic activity may alter vasa-vasorum flow that in turn influences aortic function in patients with FMV/MVP with or without MR.

It is well appreciated today that aortic function changes with age; however, age cannot be a factor in this study since patients with FMV/MVP were matched for age and gender with the control group. 28 29 30 Coronary artery disease also may affect aortic function. 16 For this reason, only patients with minimal coronary artery disease were included in this study. Moreover, the degree of coronary artery disease, though minimal, was similar between groups.

### Clinical Implications: Aorta—The Forgotten Dynamic Organ in MR

**Fig. 3:**
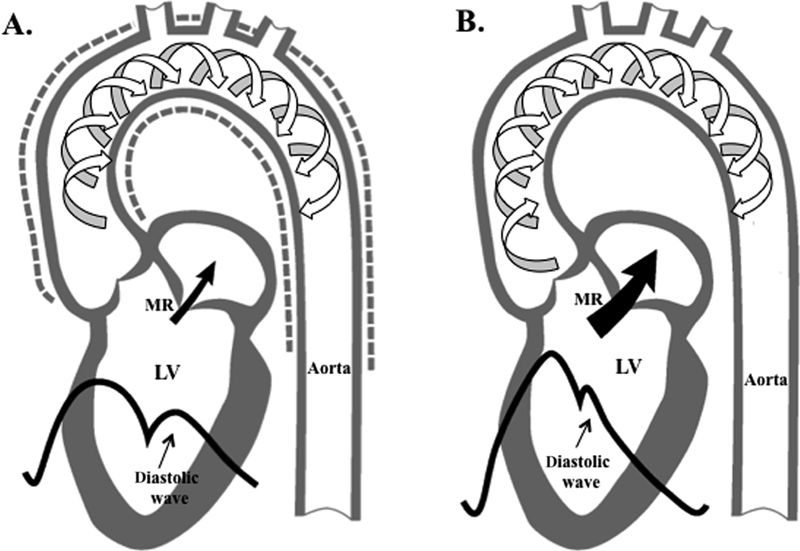
( **A** ) When blood is ejected from the left ventricle (LV) into the aorta during systole, a pulse wave is generated. The speed at which the pulse wave travels in the aorta is defined as the pulse wave velocity (PWV; white arrows) and is directly related to the elastic properties of the aortic wall (dotted line represents the expansion of a normal aorta). When the pulse wave reaches the periphery, it returns to the ascending aorta as reflected waves. Normally, the reflected waves reach the ascending aorta early in diastole, resulting in the formation of the diastolic wave that facilitates coronary blood flow. ( **B** ) In a stiff aorta, there is an increase in the PWV and reflected wave velocity; the reflected waves return early to the aortic root and fuse with the systolic portion of the pulse pressure, resulting in an increase in systolic aortic pressure and the disappearance of the diastolic wave. Increased systolic aortic pressure will increase the severity of mitral regurgitation (MR) (slightly modified from Boudoulas et al. 36 ).

Beyond serving as conduit for blood flow, the aorta is important in maintaining LV performance, myocardial perfusion, and arterial function throughout the cardiovascular system. When blood is ejected from the LV during systole into the aorta, a pulse wave is generated. The speed at which the pulse wave travels in the aorta is defined as the pulse wave velocity (PWV) and is directly related to the elastic properties of the aortic wall. Under normal conditions, when the pulse wave reaches the peripheral circulation, the waves return to the ascending aorta as reflected waves. When the elastic properties of the aorta are normal, reflected waves arrive at the root of the aorta early in diastole and form the diastolic wave that facilitates coronary blood flow. When the aorta is stiff, there is an increase in the PWV and reflected wave velocity. Thus, reflected waves arrive earlier in the root of the aorta, during systole, fusing with the systolic portion of the pulse pressure wave. This results in an increase in systolic pressure, LV work and mass, and in the disappearance of the diastolic wave. Increase in the systolic pressure may increase the severity of MR, while the disappearance of the diastolic wave may compromise coronary flow. Thus, a decrease in aortic distensibility (i.e., stiffening of the aorta) results in an increase in LV afterload, which may result in LV dysfunction, and in turn, increase in the severity of MR. Left atrial dilatation and dysfunction also ensue ( Fig. 3 ). In addition, a stiff aorta may result in impaired LV relaxation and decreased early diastolic filling, resulting in an increase in left atrial work to maintain LV stroke volume. 13 29 31 32 33 34 35 36

## Conclusion

The aorta becomes stiffer with age. It follows that aortic PWV increases progressively with age. In fact, aortic PWV doubles between the second and eighth decade of life. However, the rate of increase in the PWV is greater after the fifth decade. It should be mentioned that significant MR in patients with FMV/MVP usually occurs after the age of 50 at which the aorta becomes stiff. 28 29 30 33 Thus, abnormal aortic function, which also deteriorates with age, may play an important role in the progression of MR in patients with FMV/MVP. It follows that aortic function should be evaluated routinely in patients with FMV/MVP, especially if MR is present, in the same way that LV and left atrial structure and function are measured today.
